# The Relationship between the Testicular Blood Flow and the Semen Parameters of Rams during the Selected Periods of the Breeding and Non-Breeding Seasons

**DOI:** 10.3390/ani12060760

**Published:** 2022-03-17

**Authors:** Natalia Kozłowska, Ricardo Faundez, Kamil Borzyszkowski, Sebastian Dąbrowski, Tomasz Jasiński, Małgorzata Domino

**Affiliations:** 1 Department of Large Animal Diseases and Clinic, Institute of Veterinary Medicine, Warsaw University of Life Sciences, 02-787 Warsaw, Poland; natalia_kozlowska@sggw.edu.pl (N.K.); dabrowski.sebastian89@gmail.com (S.D.); tomasz_jasinski@sggw.edu.pl (T.J.); 2 InviMed Fertility Clinics, 02-532 Warsaw, Poland; 3 Small Animall Clinic Bokserska, 02-691 Warsaw, Poland; k.borzyszkowski@gmail.com

**Keywords:** testis, CASA, SCD, MSOME, pulse wave Doppler, ram

## Abstract

**Simple Summary:**

There is a rising demand for sheep products such as meat, wool, and milk. Within the sheep flock, rams are the most valuable animals as they represent 50% of the genetics of every sheep flock. To improve productivity, monitoring of the rams’ semen quality allows breeders to maximize fertility. This study aimed to introduce the advanced semen evaluation tests and evaluate the correlations between the parameters of the basic and advanced semen evaluation tests and the dynamics of testicular blood flow. Semen was collected from eighteen rams, and a pulse wave Doppler examination was conducted in three routine ram examination periods in Poland: before the breeding season (BBS), during the breeding season (BS), and after the breeding season (ABS). Routine and advanced semen analysis, including computer-assisted sperm analysis (CASA), sperm chromatin dispersion test (SCD), and motile sperm organelle morphology examination (MSOME), were conducted. In Doppler ultrasonography, the peak systolic velocity (PSV), end-diastolic velocity (EDV), resistive index (RI), and pulsatility index (PI) were calculated. The high-quality semen was derived during BS, whereas BBS semen demonstrated high sperm abnormalities related to sperm cells DNA fragmentation and vacuolization. The features of higher sperm abnormalities correlated with an increase in RI, PI, and EDV, respectively.

**Abstract:**

The study aimed to conduct advanced semen evaluation tests during routine ram examination periods in the breeding and non-breeding seasons and to investigate their correlation with the dynamics of testicular blood flow. Semen was collected from eighteen rams, and pulse wave Doppler examination before (BBS), during (BS), and after the breeding season (ABS). Routine and advanced semen analysis, including computer-assisted sperm analysis (CASA), sperm chromatin dispersion test (SCD), and motile sperm organelle morphology examination (MSOME), were conducted. In Doppler ultrasonography, the peak systolic velocity (PSV), end-diastolic velocity (EDV), resistive index (RI), and pulsatility index (PI) were calculated. In BS period, high sperm concentration (*p* < 0.0001) and total sperm number/ejaculate (*p* = 0.008) were noted. During the BBS period, a low percentage of forwarding motility (*p* = 0.017) and high sperm abnormalities (*p* = 0.005) were found. Also during this period, both SCD and MSOME revealed high sperm DNA fragmentation (*p* < 0.0001) and signs of vacuolization (Grade II-IV, *p* < 0.05). The advanced features of higher sperm abnormalities (Grade IV of MSOME) correlated with an increase RI (ρє <0.60;0.61>) and PI (ρє <0.46;0.52>), whereas the basic percentage of sperm abnormalities correlated with the EDV (ρє <0.44;0.73>) value. One may conclude that the current preliminary study requires further research concerning the monthly examination of a ram to provide full yearly characteristics of the relation between advanced semen evaluation tests and the dynamics of testicular blood flow.

## 1. Introduction

Classically, an evaluation of the breeding ram is carried out by physical examination of the reproductive system and an assessment of libido before the beginning of the breeding season [1]. Additional advanced methods have increased value in estimating suitability to the reproduction of rams. The breeding assessment helps to eliminate animals with hereditary defects, prevent mating with infertile animals, and control the transmission of infectious diseases [2]. Currently, several modern diagnostic methods have become available to facilitate the reproductive evaluation of the male in a field [3,4,5].

Ultrasound examination of the testes has proven to be a valuable, non-invasive technique that provides important information regarding the function and structure of testicular tissues. In recent studies, the correlations between testicular echotexture and semen quality were demonstrated in rams [6], bulls [7], dogs [8], donkeys [9], and horses [10]. Moreover, in rams, the characteristic of blood flow in the testicular artery was described [11], and the association between the testicular blood flow dynamics and the routine semen characteristics was evaluated [12]. Doppler ultrasonography became a useful tool in the assessment of vascular integrity, blood flow perfusion, and spermatogenesis in rams [12], dogs [13,14], bulls [15], and stallions [16]. It has been reported that the high metabolic activity in the seminiferous tubules in the testis, is adapted to low oxygen tension and low arterial capillary pressure. Therefore, the blood supply alterations may cause testicular dysfunction leading to male infertility [17]. Based on these findings, the principal measurement of blood flow, including peak systolic velocity (PSV), end-diastolic velocity (EDV), resistive index (RI), and pulsatility index (PI), were used in the routine clinical evaluation of fertility in humans [18] and carnivorous animals [19] as well as in ruminants [20], camelids [21], and equids [16]. 

Because male fertility is an important contributor to flock breeding potential, the detailed evaluation of semen parameters and sperm functionality has become essential. Routine semen evaluation is based on assessing parameters such as morphology, concentration, motility [22], and general assessment of testes and epididymal function. The semen examination is also more informative regarding male fertility than the basic clinical examination [23]. Specifically, sperm motility has been reported to positively correlate with the fertilizing ability of rams [24]. Recently, increasing attention has been paid to the advanced evaluation of the semen parameters using computer-assisted sperm analysis (CASA) [25,26], sperm chromatin dispersion test (SCD) [27,28], or motile sperm organelle morphology examination (MSOME) [29,30]. The application of CASA allows us to quantify a wide range of sperm movement patterns and provides more detailed and accurate results than manual microscopic observation [31]. This system has been widely used in human [32] and veterinary medicine [33], including ram semen evaluation [20,34]. CASA provides motility and velocity data to correlate with sperm penetration assay and assessment of fertility potential [32,35]. Curvilinear velocity (VCL) and average path velocity (VAP) from CASA were described as positively correlated to the ability of sperms to migrate in ewe cervical mucus [36]. On the other hand, the SCD test has been suggested as a complementary parameter of sperm quality. The SCD test allows determining sperm DNA fragmentation index (DFI) and provides information after oocyte fertilization of the developmental potential of embryos. An increased DFI has been associated with male infertility [37]. The SCD test has been applied in ruminants [38,39] including rams [40], carnivorous animals [41], and equids [40,42,43]. Moreover, the use of MSOME provides high-quality data of sperm morphology which is crucial in the prediction of fertilizing capacity [29]. MSOME is a real-time technique that enables assessing morphological changes in sperm organelles at high optical magnification. MSOME has been used to select normal morphological sperm to improve the pregnancy rate in humans [44]. The high magnification technique (6600×) allows detecting vacuole-like changes in the head, considered defects associated with abnormal chromatin packaging or fragmentation of sperm DNA [45]. Poor semen morphology has been reported as an indicator of decreased fertility in men [22] and domestic animals [46,47]. Computer morphometric analysis [48,49] contributes to assessing the fertilization potential of semen. However, because CASA and MSOME could represent improvements in routine sperm analysis with potential clinical implications, no data are available from MSOME application in rams.

Due to the association between changes in monthly dynamics of testicular blood flow, testicular volume, plasma hormone concentrations, and the routine semen characteristics in rams, a detailed advanced assessment during the breeding and non-breeding season was required. We hypothesized the association between season, Doppler and semen parameters. Therefore, this study aimed to conduct the hemodynamic parameters of testicular blood flow and advanced semen evaluation tests, CASA, SCD, MSOME, of ram’s semen in the selected seasons.

## 2. Materials and Methods

### 2.1. Animals

Eighteen Polish Heath rams aged from 3 to 5 years, weighing 45–50 kg, underwent the standard veterinary clinical examinations. Rams were maintained in the sheep herd of the Warsaw University of Life Sciences. Rams were housed with the same management and were fed twice a day with grass hay (crude protein 10%) and commercial concentrate (crude protein 14%) introduced to the diet 4 weeks before breeding season. Animals had free access to mineralized salt and water. The body condition score was maintained between 3 and 4. Rams were routinely vaccinated and received treatments for internal parasites. Natural mating was introduced with a ram-to-ewe ratio of 1:17. Rams were housed under natural daylight, temperature, and relative humidity. 

The standard veterinary clinical examination was conducted during the periods of routine ram examination in the herd, before the breeding season (BBS, September), during the breeding season (BS, November), and after the breeding season during the sheep lambing period (ABS, April). Daily sunny hours were obtained from the Institute of Meteorology and Water Management, National Research Institute, Warsaw, Poland, whereas temperature and relative humidity were measured daily. The mean values for the selected examined months were (i) in September (mean sunny hours: 13 h; ambient temperature: 15 ± 1.4 °C; humidity: 51 ± 1.3%); in November (mean sunny hours: 9 h; ambient temperature: 5 ± 0.9 °C; humidity: 63 ± 1.1%), and in April (mean sunny hours: 13 h; ambient temperature: 11 ± 1.8 °C; humidity: 46 ± 1.0%). In each of the given months, the rams were examined three times with an interval of one week. Each examination included a firstly ultrasonographical examination of the testes with the examination of hemodynamic parameters of the testicular artery, and secondly the semen collection.

Before the study, the rams were routinely vaccinated and dewormed. Before each examination, rams underwent basic clinical examination, which included the following: rectal temperature, heart rate, respiratory rate, capillary refill time test, mucous membranes, and lymph nodes evaluation. All rams showed normal ranges of temperature (38.5–39.0 °C), heart rate (60–90 beat/min), respiratory rate (16–30 breath/min), capillary refilling time test (1–2 s), and no pathological changes of mucous membranes and lymph nodes during the whole length of the study period. Detailed examination of the penis and prepuce, size, symmetry, consistency of both testes, and the course of the spermatic cord was evaluated. All rams experienced no pathological signs during the whole length of the study period.

### 2.2. Semen Collection and Evaluation

**Figure 1:**
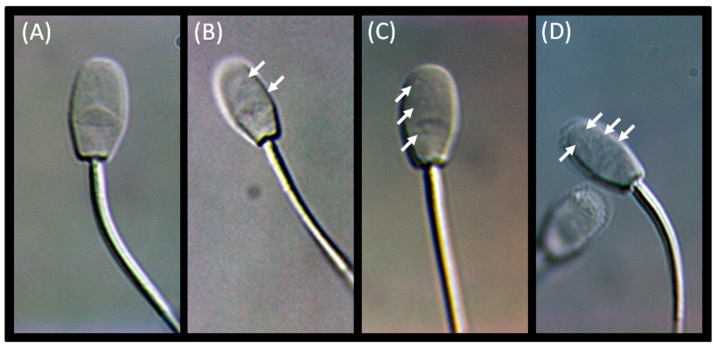
Microscopic images of ram sperm cells under high optical magnification (6.000–10.000×). Sperm cells represented four grades of motile sperm organelle morphology examination (MSOME): Grade I (**A**); Grade II (**B**); Grade III (**C**); Grade IV (**D**). Cytoplasmic vacuoles were marked with white arrows.

Semen samples were obtained into an artificial vagina dedicated to rams (short outer casing, small inside diameter, water temperature: 40–41 °C). Immediately after collection, the ejaculate volume (mL) was measured using calibrated semen collection tube. The routine sperm analysis was conducted following Evans’s and Maxwell’s protocol used in Hedia et al. [12]. The sperm concentration (×10^9^/mL), total sperm number per ejaculate (×10^9^), forward motility (%), sperm vitality (%), and morphological sperm abnormalities (%) were evaluated. The advanced semen evaluation, including CASA, SCD, and MSOME tests, was conducted following recent protocols [20,40,50], respectively. The Sperm Class Analyzer CASA system (Microptic S.L, Veterinary, Scientific Pack, Barcelona, Spain) was used. The total and forward motility and specific parameters such as curvilinear velocity (VCL) (µm/sec), straight-line velocity (VSL) (µm/sec), average path velocity (VAP) (µm/sec), linearity (LIN) (%), straightness (STR) (%), wobble (WOB) (%) were measured. Then, the spermatozoa were classified into Slow Default (SD), Medium Default (MD), and Rapid Default (RD). According to the manufacturer’s instructions, DNA fragmentation was evaluated using the Halomax^®^ kit (Halotech DNA SL, Madrid, Spain), and the DNA fragmentation index (DFI) was calculated. Sperm morphology examination was performed using the morphology module of CASA. The MSOME test was conducted using an inverted microscope DMi8, Leica, Germany, equipped with DIC/Nomarski optics using a Leica objective HCX PL FLUOTAR 100×/1.30, under oil immersion. A microdroplet of semen suspension in SWM medium (FujiFilm Irvine Scientific, Santa Ana, CA, USA) was placed in a sterile glass dish (FluoroDish, World Precision Instruments, Sarasota, FL, USA) under paraffin oil (NidOil, Nidacon, Brussels, Belgium). The examination was performed with Leica DMI 6000B inverted microscope. The sperm cells were observed at high magnification (6.000–10.000×). At least 200 sperm were examined for each sample, and photo documentation was performed. The spermatozoa were classified into four grades according to Vanderzwalmen et al. [50] including the following: Grade I: normal form and no vacuoles; Grade II: normal form and ≤2 small vacuoles; Grade III: normal form and >2 small vacuoles or at least one large vacuole; Grade IV: large vacuole and abnormal head shapes or other abnormalities. Images visualized sperm cells representing four grades of MSOME were presented in Figure 1.

### 2.3. Ultrasound Examination and Doppler Application

Ultrasound examinations were performed at a fixed time (12 A.M.), after morning feeding in each season. First, all the animals have undergone a clinical examination then after some rest (assessed by breath rate), one by one were taken for ultrasound examination to reduce the stress and potential effect on testicular artery blood flow. Rams were restrained by an assistant in a standing position without any sedation. The scrotal skin was shaved, cleaned, and covered with ultrasonographic gel to facilitate imaging. For each ram, both testes were evaluated by the same operator. Ultrasonographic imaging of testicles and testicular blood flow was carried out using a linear array of 3–13 MHz (MyLab Alpha, Esaote, Genoa, Italy). Initially, the probe was placed on the caudal surface of the testicle along the longitudinal axis and was moved to examine the testicular parenchyma. Then, the probe was pushed upwards to image vessels of the spermatic cord. Color Doppler assessment was performed after localization of the largest observable section of the testicular artery. The ultrasound settings (focus, gains, brightness, and contrast) were standardized, and the same settings were used for all the examinations.

**Figure 2:**
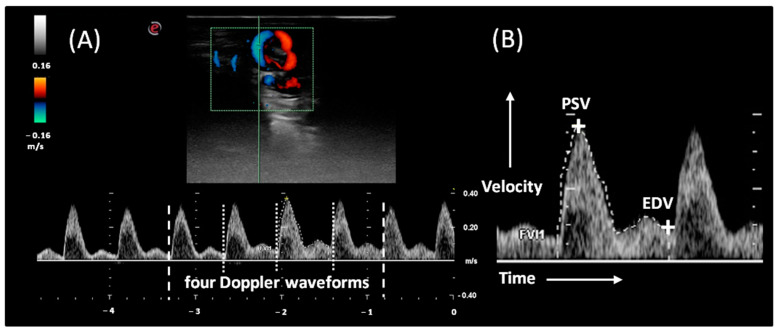
Schematic view of the blood flow measures. The pulse wave Doppler ultrasound images of the testicular artery in the spermatic cord (**A**) with a single Doppler waveform (dropped line separation) formed four Doppler waveforms selected for evaluation (dashed line separation). Sample Doppler waveform with marked the peak systolic velocity (PSV) and end-diastolic velocity (EDV) (**B**).

The testicular artery was visualized as blue or red areas caused by the blood flow towards and away from the probe. The vessel was possibly imaged in a sagittal or oblique section. The Doppler spectra cursor was placed on the testicular artery lumen, and pulse wave spectral Doppler was introduced. The gate was kept constant at 2 mm, the angle of insonation was set at 0°, and the baseline was lowered. The pulse repetition frequency was set at 2.9 kHz. A minimum of four Doppler waveforms were measured. Differentiation between the testicular artery and testicular vein was based on a spectral graph. The peak systolic velocity (PSV) and end-diastolic velocity (EDV) of the testicular artery were measured, and the resistive index (RI = [PSV−EDV]/PSV) and pulsatility index (PI = [SPV−EDV]/mean velocity) were calculated by the machine according to the formula (Figure 2).

### 2.4. Statistical Analyses

Univariate marginal distributions of the semen parameters and the testicular blood flow were tested independently for given periods using a Shapiro–Wilk test. The comparison between given periods was assessed by a one-way analysis of variance (ANOVA) followed by Tukey’s multiple comparisons test for data series showing Gaussian distribution; or the Kruskal–Wallis test followed by Dunn’s multiple comparisons test for non-Gaussian data. The significance level was established as *p* < 0.05. Numerical data were presented as the mean ± standard deviation (SD) supported with the coefficient of variation (CV%). Spearman’s rank correlation coefficients (ρ) among blood flow parameters and the semen quality parameters were calculated independently for the given periods. Spearman’s rank correlation coefficients were considered significant for *p* < 0.05. All statistical analyses were performed using GraphPad Prism6 software (GraphPad Software Inc., San Diego, CA, USA).

## 3. Results

### 3.1. Routine Semen Evaluation Test

The results of the routine semen evaluation test were presented in Table 1. No differences in mean ejaculate volume (*p* = 0.630) and percent of sperm vitality (*p* = 0.104) were noted between given periods. However, a lower sperm concentration value (*p* < 0.0001) and total sperm number per ejaculate (*p* = 0.008) were noted BBS and ABS, than during BS. Moreover, a lower percentage of forwarding motility (*p* = 0.017) and higher sperm abnormalities (*p* = 0.005) were found BBS than during BS, with no differences ABS.

### 3.2. Advanced Semen Evaluation Tests

The parameters of the advanced semen evaluation tests, CASA, SCD, and MSOME, were summarized in Table 2 and Table 3, respectively.

Concerning CASA results, sperm motility characteristic was presented in Table 2. Significant differences between given periods were found only within the curvilinear velocity of SD and MD motility sperm. A lower VCL values were obtained BBS and during BS than ABS, when both SD fraction (*p* = 0.015) and MD fraction (*p* = 0.003) were concerned. No other differences (*p* > 0.05) in sperm motility characteristics were found between given periods.

Concerning SCD and MSOME results, the features of the sperm head abnormalities were presented in Table 3. The percentage of sperm cells with fragmented DNA (DFI) (*p* < 0.0001) as well as the percentage of abnormal sperm cells (Grade II, *p* = 0.0006; Grade III, *p* = 0.004; Grade IV, *p* < 0.0001) were higher BBS compare to BS and ABS. On the contrary, the percentage of sperm cells without signs of vacuolization (Grade I, *p* < 0.0001) was lower BBS than in BS and ABS.

### 3.3. Ultrasound Examination and Doppler Application

**Figure 3:**
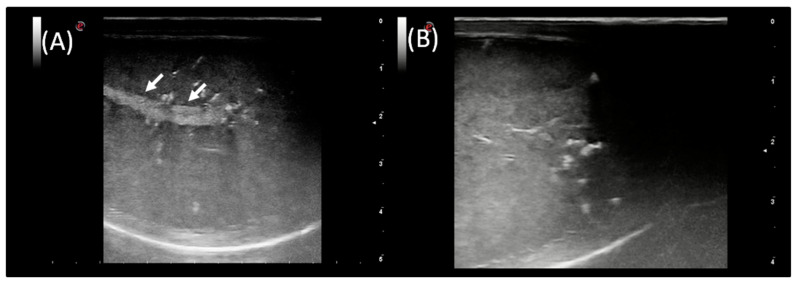
Gray-scale ultrasound images of testicular parenchyma in sagittal view (**A**) and longitudinal view (**B**) in the ram. The hyperechogenic mediastinum testis was marked with white arrows.

In the standard B-mode assessment of both testes of each ram, the parenchyma appeared homogeneous with central linear hyperechogenic mediastinum testis. The echogenicity and size of mediastinum testis varied between animals. There were no differences in testicular echotexture between images obtained from left and right testes (Figure 3). No clinical and ultrasonographical abnormalities were present in any of the rams included in this investigation.

There were no significant differences between the right and left testes concerning Doppler parameters of the testicular arteries (PSV, EDV, RI, and PI). Thus, the means of the right and left testes were used for further analysis. There were no significant differences between the first, second, and third examinations in the given months concerning Doppler parameters of the testicular arteries.

The waveforms of the testicular artery were characteristic of a monophasic blood flow pattern with a short systolic flow followed by a long diastolic decrement. Doppler parameters of the testicular arteries were shown in Table 4.

A higher PSV value (*p* < 0.0001) was noted BBS than BS and ABS, whereas a higher EDV value (*p* < 0.0001) was recorded BS than BBS and ABS. Both RI and PI values were the highest BBS, the lowest BS, and on the middle level ABS (*p* < 0.0001).

### 3.4. Associations between the Semen Parameters and the Testicular Blood Flow

As depicted in Table 5, positive correlations between PSV and ejaculate volume and negative correlations between PSV and sperm vitality were noted as moderate during BS and strong ABS, with no significant correlation BBS. The weak negative correlations between PSV and grade IV of MSOME as well as between EDV and grade IV of MSOME were observed only during BS. Positive correlations from weak to strong in all given periods were observed between EDV and total sperm number per ejaculate as well as between EDV and sperm abnormalities. Negative moderate correlations were found between the following: RI and sperm concentration BBS and ABS; RI and curvilinear velocity of SD fraction BBS and during BS; RI and linearity, straightness, and wobble of RD fraction only during BS. Moreover, moderate positive correlations were noted between RI and grade IV of MSOME, as well as PI and grade IV of MSOME BBS and ABS but not during BS. Finally, strong and moderate negative correlations were observed between PI and forward motility and sperm concentration, respectively, similarly BBS and ABS but not during BS. No other significant correlation between hemodynamic parameters of the testicular artery and the routine and advanced semen measures was found in the periods of routine ram examination in the herd.

## 4. Discussion

It is well established that in the ram, sexual activity is subject to seasonal variations, the intensity of which depends on breed and latitude [51]. These variations can affect all the components of the reproductive function, particularly the gonads’ characteristics, quality of the semen, and its fertilizing ability [1]. Monthly and seasonal changes in testicular blood flow with semen characteristics over 12 months in the ram have been presented for the first time by Hedia et al. [12], and the highest variations were noted just before, during, and after the breeding season. As the routine examination of ram in the Polish herds is not carried out every month, but only during designated control periods [52,53], the current preliminary study broadened the routine semen analysis into advanced techniques during these key periods for the sheep herd reproductive inspection. However, it may be noted that further monthly research on the relation between advanced semen evaluation tests and the dynamics of testicular blood flow is required to provide full yearly characteristics.

In the present preliminary study, semen analysis indicated that total semen volume had no significant differences and correlations within the concerned periods. The highest semen volume in spring and the lowest in winter were reported in Algeria [54] and Greece [55], whereas the lowest semen volume in spring and the highest in autumn was confirmed in Egypt [12], Hungary [56], and Spain [57]. One may observe, in the seasonal livestock animals such as sheep [1,51], that the region of the world is crucial for the seasonal reproductive variations. Therefore, to avoid inaccuracies, it is better to use the term before, during, and after the breeding season, as they vary between regions of the world. Moreover, the results of the semen evaluation and the dynamics of testicular blood flow might be affected by the individual characteristics of each animal [11], the technology used in semen collection [58], environmental conditions, and the breed [34]. The current results of the other routine semen parameters, such as sperm concentration, total sperm number per ejaculate, and forward motility were in agreement with the values characteristic for the periods in BBS, BS, and ABS periods, when the monthly evaluation was performed [12]. In both, the previous [12] and the present studies, the highest values were described during BS and the lowest during BBS. Furthermore, in both studies, no significant changes in sperm vitality were found [12].

In the current study, the percentage of sperm abnormalities, the percentage of fragmented DNA and the grades of vacuolization in MSOME (grade II–IV) were the highest BBS than in other examined periods, which points to potentially decreased fertility. The high percentage of sperm abnormalities during BBS was in line with the Hedia et al. report [12]; however, the MSOME results in rams have not been published recently. Foxcroft et al. [59] evidenced the positive correlations between the integrity of sperm chromatin and semen motility and morphology. Vicente-Fiel et al. [60] reported that rams with high fertility exhibited significantly lower rates of sperm DNA fragmentation (DFI) in SCD test, than spermatozoa from low fertility groups. Moreover, Malama et al. [61] suggested that the scrotal temperature might interfere with the process of sperm chromatin stabilization in the epididymis. The impact of temperature on the semen quality and testosterone blood serum concentration was confirmed by Hedia et al. [12] and Alves et al. [62]. Therefore, in the current research, rams in the period BBS, which in Poland occurs right after the warm summer months [52,53], may be subjected to an increase in temperature affecting the sperm quality. Specifically, in the period ABS, when the ambient temperature (11 ± 1.8 °C) was comparable to BBS (15 ± 1.4 °C), no similar results were noted. Such an explanation is consistent with Malama et al.’s [61] suggestion that the high summer temperatures’ seasonality affects the ram semen DNA stability. However, these hypotheses require further research on the monthly model, considering both the MSOME rams’ ejaculate evaluation and the surface scrotal temperature examination. To the best of our knowledge, this is the first report describing the MSOME assessment in rams; the surface scrotal temperature was previously examined using infrared thermography both in bulls [63] as well in rams [62]. 

In the current study, the results of the advanced semen evaluation tests, SCD and MSOME, indicated the increased sperm quality during BS, which is in agreement with the previous reports [12,55,56,57]. However, the sperm motility characteristics in CASA evaluation did not differ between given periods, excepting VCL of slow and medium default fractions. Although in the previous research three sperm motility subpopulations were also investigated [64], there are no data on seasonal variations of CASA parameters between the periods of routine ram examination in the herd as well as monthly changes in the breeding season [34,60,64]. Kumar et al. [34] examined the semen of Malpura ram lambs housed under the intensive management system to find the age when ram lambs are able to produce good quality semen. Vicente-Fiel et al. [60] compared the semen of the high and low fertility Rasa Aragonesa rams to indicate the differences in several sperm quality parameters and provide the possibility of the selection of rams for artificial insemination based on sperm quality data. Santolaria et al. [64] also examined the semen of Rasa Aragonesa rams to find CASA parameters useful for a predictive capacity on field fertility after artificial insemination. One may observe that the values of CASA parameters of rapid default fraction were similar in the current study and the Vicente-Fiel et al. [60] and Santolaria et al. [64] research, whereas Kumar et al. [34] reported higher motility results, which may result from examining rams of different breeds. One may carefully suggest, the highest percentage of sperm abnormalities reported here and in the previous study [12] during BBS may result from the changes in the sperm morphology and abnormal chromatin packaging or the fragmentation of sperm DNA [45] rather than insufficient spermatozoa capacity to migration in the female reproductive tract [36]. However, monthly research is required to better determine the relations between sperm abnormalities, SCD, MSOME, and CASA parameters in Polish rams over the yearling seasons.

In the current study, the blood flow measures did not differ between the right and left testis, similarly to previous research [12], indicating the symmetry which is essential in a clinical examination of a ram’s fertility [65]. Both RI and PI values were the highest BBS, the lowest during BS, and on the middle-level ABS, which is in agreement with the Hedia et al. [12] report. Hedia et al. [12] have suggested that an increase in vascular resistance BBS, after the hottest months, may be an indicator of decreased blood flow and semen quality. Carvajal-Serna et al. [66] reported that RI and PI were inversely linked to the blood-flow perfusion, suggesting that an increase in blood flow in the testis would increase the number of cells within the seminal tubule, thus reducing the lumen area. In men, RI decreased when the inflammatory process develops and increases with the progression of the degenerative processes [67,68]. Moreover, higher RI values were positively correlated with a higher percentage of abnormal sperm cells [69], although in the current study in rams no correlation was found between RI and the percentage of sperm abnormalities. A positive correlation between RI and grade IV of MSOME, resulting from severe vacuolization [29,44], was noted. Moreover, during BS, negative correlations were found between RI and CASA-returned curvilinear velocity, linearity, straightness, and wobble, similarly to Hedia et al. [12]. However, in Hedia et al. [12], the routine sperm cell motility evaluation rather than the detailed computer-assisted motility analysis was performed. In contrast, no similar correlations were observed in previous studies in rams [11,20]. However, in stallions [70] and bulls [15], the negative correlation between RI of the testicular artery and the routine sperm motility parameters was also reported. In dogs, increased RI and PI were suggested as an important factor affecting spermatogenesis [71]. This may be supported in the current study by a positive correlation between PI and grade IV of MSOME as well negative correlations between PI and forward motility and sperm concentration, respectively. In the current study, the positive correlations between blood flow measurements and sperm abnormalities were found in all given periods; however, in EDV rather than PI or RI. Because EDV value has not been investigated in the Hedia et al. [12] and Ntemka et al. [20] studies, the correlations observed here required confirmation in rams’ semen examined monthly over the yearling season. 

## 5. Conclusions

Both basic and advanced semen evaluation tests of ram’s semen indicated higher sperm abnormalities during BBS than during BS and ABS when the periods of routine ram examination in the herd were considered. Based on CASA, SCD, and MSOME results, these abnormalities may result from sperm DNA fragmentation and sperm head vacuolization rather than insufficient spermatozoa motility capacity. Specifically, it may be suggested that the advanced features of higher sperm abnormalities correlated with an increased RI and PI measurements of the testicular blood flow, whereas the basic percentage of sperm abnormalities correlated with the EDV value. These preliminary results indicate that further monthly research is required to precisely determine the association between advanced semen evaluation tests and the dynamics of testicular blood flow, which has been highlighted in the present work. Currently, several modern diagnostic methods have become available to facilitate the reproductive evaluation of the male in a field, the introduction of the advanced methods into the routine ram examination in the herd will improve the estimating suitability to the reproduction of rams. 

## Figures and Tables

**Table 1:** The measures (mean ± SD (CV%)) of routine semen evaluation tests in the selected periods of routine ram examination in the herd, before the breeding season (BBS), during the breeding season (BS), and after the breeding season (ABS).

	Breeding Season			*p* Value
	BBS	BS	ABS	
Ejaculate volume (mL)	0.48 ± 0.22 ^a^ (45.9)	0.56 ± 0.39 ^a^ (68.9)	0.44 ± 0.27 ^a^ (61.5)	0.630
Sperm concentration (×10^9^/mL)	1.43 ± 0.54 ^a^ (37.9)	3.21 ± 0.75 ^b^ (23.5)	1.97 ± 0.93 ^a^ (47.2)	<0.0001
Total sperm number per ejaculate (×10^9^)	0.66 ± 0.32 ^a^ (47.8)	1.88 ± 1.42 ^b^ (75.7)	0.84 ± 0.73 ^a^ (86.9)	0.008
Forward motility (%)	54.1 ± 14.2 ^a^ (26.2)	72.0 ± 17.1 ^b^ (23.7)	67.6 ± 12.9 ^ab^ (19.1)	0.017
Sperm vitality (%)	66.7 ± 13.6 ^a^ (20.5)	77.4 ± 15.2 ^a^ (19.64)	76.2 ± 21.0 ^a^ (27.5)	0.104
Sperm abnormalities (%)	18.5 ± 9.3 ^a^ (49.9)	7.3 ± 6.8 ^b^ (93.6)	11.3 ± 6.5 ^ab^ (57.7)	0.005

Significant differences between given months were depicted by letters (^a^, ^b^; for *p* < 0.05).

**Table 2:** Sperm motility characteristics (mean ± SD (CV%)) of advanced semen evaluation test, computer-assisted sperm analysis (CASA), in the selected periods of routine ram examination in the herd, before the breeding season (BBS), during the breeding season (BS), and after the breeding season (ABS).

		Breeding Season			*p* Value
		BBS	BS	ABS	
VCL (µm/s)	SD	22.9 ± 9.32 ^a^ (40.6)	26.6 ± 15.5 ^a^ (58.0)	40.1 ± 14.6 ^b^ (36.5)	0.015
	MD	44.1 ± 20.4 ^a^ (46.2)	53.5 ± 15.9 ^a^ (29.7)	63.3 ± 4.5 ^b^ (7.0)	0.003
	RD	120.3 ± 41.3 ^a^ (34.3)	110.1 ± 15.7 ^a^ (14.2)	114.8 ± 12.9 ^a^ (31.5)	0.664
VSL (µm/s)	SD	9.2 ± 4.2 ^a^ (46.1)	10.1 ± 6.7 ^a^ (66.5)	14.0 ± 5.9 ^a^ (42.7)	0.201
	MD	30.3 ± 29.8 ^a^ (98.4)	26.5 ± 13.9 ^a^ (52.5)	25.9 ± 9.2 ^a^ (35.5)	0.831
	RD	81.1 ± 46.5 ^a^ (57.4)	72.9 ± 24.9 ^a^ (34.2)	58.9 ± 13.4 ^a^ (35.5)	0.355
VAP (µm/s)	SD	14.2 ± 5.9 ^a^ (42.1)	18.8 ± 13.6 ^a^ (72.4)	24.1 ± 9.3 ^a^ (38.5)	0.098
	MD	32.2 ± 15.1 ^a^ (46.9)	36.4 ± 15.0 ^a^ (41.4)	39.8 ± 7.9 ^a^ (19.9)	0.489
	RD	94.0 ± 48.6 ^a^ (51.7)	87.0 ± 20.1 ^a^ (23.1)	80.5 ± 15.9 ^a^ (19.7)	0.773
LIN (µm/s)	SD	38.0 ± 19.6 ^a^ (51.5)	37.1 ± 16.3 ^a^ (44.0)	34.9 ± 8.2 ^a^ (23.5)	0.837
	MD	46.2 ± 23.1 ^a^ (49.9)	46.5 ± 20.3 ^a^ (43.6)	41.0 ± 14.6 ^a^ (35.7)	0.505
	RD	64.2 ± 22.8 ^a^ (35.5)	65.3 ± 18.4 ^a^ (28.1)	51.4 ± 18.5 ^a^ (36.1)	0.221
STR (µm/s)	SD	59.4 ± 24.1 ^a^ (40.5)	63.1 ± 13.7 ^a^ (21.7)	57.8 ± 7.7 ^a^ (13.3)	0.630
	MD	64.1 ± 28.2 ^a^ (44.0)	68.0 ± 18.6 ^a^ (27.3)	63.8 ± 10.4 ^a^ (16.3)	0.382
	RD	79.6 ± 16.8 ^a^ (21.2)	81.7 ± 13.5 ^a^ (16.7)	71.3 ± 13.4 ^a^ (18.9)	0.152
WOB (µm/s)	SD	57.1 ± 20.9 ^a^ (36.6)	57.3 ± 15.3 ^a^ (26.7)	59.9 ± 7.5 ^a^ (12.6)	0.692
	MD	68.5 ± 24.8 ^a^ (36.3)	65.1 ± 19.9 ^a^ (26.1)	62.8 ± 11.7 ^a^ (18.6)	0.126
	RD	77.9 ± 17.6 ^a^ (22.6)	78.2 ± 12.0 ^a^ (15.3)	70.1 ± 12.8 ^a^ (18.2)	0.240

Significant differences between given months were depicted by letters (^a^, ^b^; for *p* < 0.05). VCL, the curvilinear velocity; VSL, the straight-line velocity; VAP, average path velocity; LIN, linearity; STR, straightness; WOB, wobble; RD, rapid default; MD, medium default; SD, slow default.

**Table 3:** The measures (mean ± SD (CV%)) of advanced semen evaluation tests, sperm chromatin dispersion test (SCD) and motile sperm organelle morphology examination (MSOME), in the selected periods of routine ram examination in the herd, before the breeding season (BBS), during the breeding season (BS), and after the breeding season (ABS).

	Breeding Season			*p* Value
	BBS	BS	ABS	
DFI (%)	3.26 ± 0.64 ^a^ (19.7)	1.62 ± 0.53 ^b^ (32.6)	2.13 ± 1.06 ^b^ (49.8)	<0.0001
Grade I (%)	91.6 ± 2.48 ^a^ (2.7)	96.9 ± 2.36 ^b^ (2.4)	96.2 ± 2.25 ^b^ (2.3)	<0.0001
Grade II (%)	4.21 ± 0.96 ^a^ (22.8)	2.10 ± 1.07 ^b^ (51.3)	2.81 ± 1.53 ^b^ (54.5)	0.0006
Grade III (%)	2.51 ± 1.47 ^a^ (58.7)	0.82 ± 0.77 ^b^ (92.1)	0.90 ± 0.88 ^b^ (97.6)	0.004
Grade IV (%)	1.66 ± 0.94 ^a^ (56.5)	0.19 ± 0.10 ^b^ (98.8)	0.11 ± 0.10 ^b^ (98.8)	<0.0001

Significant differences between given months were depicted by letters (^a^, ^b^; for *p* < 0.05). DFI, sperm DNA fragmentation index.

**Table 4:** The measures (mean ± SD (CV%)) of the blood flow measures in the testicular artery in the selected periods of routine ram examination in the herd, before the breeding season (BBS), during the breeding season (BS), and after the breeding season (ABS).

	Breeding Season			*p* Value
	BBS	BS	ABS	
PSV (cm/s)	37.9 ± 11.0 ^a^ (28.8)	27.3 ± 6.51 ^b^ (23.8)	31.0 ± 6.65 ^b^ (21.5)	<0.0001
EDV (cm/s)	7.16 ± 2.95 ^a^ (41.2)	9.90 ± 2.88 ^b^ (29.1)	7.52 ± 2.22 ^a^ (28.6)	<0.0001
RI	0.81 ± 0.06 ^a^ (7.5)	0.61 ± 0.09 ^b^ (14.6)	0.75 ± 0.05 ^c^ (6.3)	<0.0001
PI	1.82 ± 0.35 ^a^ (19.5)	1.03 ± 0.26 ^b^ (25.3)	1.58 ± 0.25 ^c^ (15.8)	<0.0001

Significant differences between given months were depicted by letters (^a^, ^b^, ^c^; for *p* < 0.05). PSV, the peak systolic velocity; EDV, the end-diastolic velocity; RI, the resistive index; PI, pulsatility index.

**Table 5:** Spearman correlation coefficients (ρ) between the blood flow measures in the testicular artery and parameters of routine and advanced semen evaluation tests in the selected periods of routine ram examination in the herd, before the breeding season (BBS), during the breeding season (BS), and after the breeding season (ABS).

Blood Flow	Semen Parameters	Breeding Season		
		BBS	BS	ABS
PSV	Ejaculate volume		0.61; *p* = 0.032	0.76; *p* = 0.009
PSV	Sperm vitality		−0.64; *p* = 0.042	−0.70; *p* = 0.019
PSV	MSOME Grade IV		−0.40; *p* = 0.024	
EDV	MSOME Grade IV		−0.38; *p* = 0.038	
EDV	Total sperm number per ejaculate	0.76; *p* < 0.0001	0.54; *p* = 0.046	0.88; *p* = 0.0007
EDV	Sperm abnormalities	0.73; *p* = 0.030	0.44; *p* = 0.0003	0.48; *p* = 0.020
RI	Sperm concentration	−0.61; *p* = 0.002		−0.68; *p* = 0.024
RI	CASA VCL SD	−0.65; *p* = 0.023	−0.63; *p* = 0.039	
RI	CASA LIN RD		−0.69; *p* = 0.024	
RI	CASA STR RD		−0.67; *p* = 0.029	
RI	CASA WOB RD		−0.66; *p* = 0.030	
RI	MSOME Grade IV	0.61; *p* = 0.034		0.60; *p* = 0.036
PI	Sperm concentration	−0.68; *p* = 0.001		−0.66; *p* = 0.027
PI	Forward motility	−0.74; *p* < 0.0001		−0.70; *p* = 0.016
PI	MSOME Grade IV	0.52; *p* = 0.024		0.46; *p* = 0.018

The Spearman correlation coefficient with significant level (ρ; p) were considered significant for *p* < 0.05. PSV, the peak systolic velocity; EDV, the end-diastolic velocity; RI, the resistive index; PI, pulsatility index; MSOME, motile sperm organelle morphology examination; CASA, computer-assisted sperm analysis; VCL, the curvilinear velocity; LIN, linearity; STR, straightness; WOB, wobble; SD, slow default; RD, rapid default.

## Data Availability

The data presented in this study are available on request from the corresponding author.
